# Efficiency of inorganic fungicides against the formation of *Erysiphe necator* chasmothecia in vineyards

**DOI:** 10.1002/ps.7487

**Published:** 2023-04-14

**Authors:** Stefan Möth, Markus Redl, Silvia Winter, Florian Hüttner, Siegrid Steinkellner

**Affiliations:** ^1^ Department of Crop Sciences Institute of Plant Protection, University of Natural Resources and Life Sciences, Vienna, Gregor‐Mendel‐Strasse 33 Vienna Austria

**Keywords:** powdery mildew, sulphur, copper, viticulture, organic, pest management

## Abstract

**BACKGROUND:**

A reduction in chasmothecia, an important inoculum of grape powdery mildew (*Erysiphe necator* Schwein.), is essential for disease control in vineyards; the use of fungicides during the formation of chasmothecia on vine leaves, late in the growing season, may accomplish this. Inorganic fungicides, such as sulphur, copper, and potassium bicarbonate, are very useful for this purpose because of their multisite mode of action. The aim of this study was to evaluate chasmothecia reduction using different fungicide applications late in the growing season in commercially managed vineyards and in an exact application trial.

**RESULTS:**

Chasmothecia on vine leaves were reduced in commercial vineyards by four copper (*P* = 0.01) and five potassium bicarbonate (*P* = 0.026) applications. The positive effect of potassium bicarbonate was also confirmed in the application trial, where two applications showed lower chasmothecia numbers than the control (*P* = 0.002).

**CONCLUSION:**

The application of inorganic fungicides reduced the amount of chasmothecia as the primary inoculum source. Potassium bicarbonate and copper are of further interest for disease control as these fungicides can be used by organic and conventional wine growers. The application of these fungicides should be carried out as late as possible before harvest to reduce chasmothecia formation and, consequently, the potential for powdery mildew infestation in the subsequent season. © 2023 The Authors. *Pest Management Science* published by John Wiley & Sons Ltd on behalf of Society of Chemical Industry.

## Graphical abstract

Densities of chasmothecia from grape powdery mildew (*Erysiphe necator*) were significantly reduced by inorganic fungicides in commercial vineyards. Late applications of potassium bicarbonate were especially efficient.

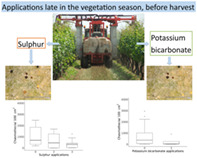

## 1 INTRODUCTION

Powdery mildew on grapes is one of the most widespread diseases in grapevines and can be highly damaging. The disease can cause crop losses of up to 100% and especially reduces grape and wine quality.^1^ The causal agent of the disease, *Erysiphe necator* Schwein. (syn*. Uncinula necator* (Schwein.) Burrill), can perennate either as chasmothecia‐containing ascospores^2^ or latently as mycelia in the buds of the vines.^3^ Primary infections of the leaves in spring can therefore be caused by ascospores from chasmothecia^2^, ^4^ or conidia from shoots coated with mycelia originating in infected buds.^5^

Globally, chasmothecia are the most important source of inoculum.^2^, ^4^, ^6^, ^7^, ^8^, ^9^, ^10^, ^11^ Their formation starts on grapevine tissue at the end of summer. During the morphological maturation process,^12^ they change their colour through melanisation, starting from white after initiation, to yellow, then brown, finally appearing black.^12^, ^13^ Chasmothecia are dispersed by rain to the exfoliating bark of vines and onto the soil,^12^ or they remain on the leaves in the absence of rain.^6^ In most wine‐growing regions, only chasmothecia overwintering on the exfoliating bark contain a high number of viable ascospores after bud break.^4^, ^12^ Furthermore, chasmothecia can only release ascospores after a physiological, temperature‐influenced maturation process.^14^, ^15^ More than 480  degree‐days (base 8 °C) from the beginning of chasmothecia formation are needed for the first potential ascospore release.^15^ The percentage of total released ascospores per season is influenced by temperature (accumulated degree‐days based on 0 °C, starting on 1 January) and by potential discharge events (days with more than 2.5 mm precipitation and a daily maximum temperature above 0 °C).^14^ Most of the ascospores are released between bud burst and bloom.^7^, ^15^, ^16^

The number of chasmothecia on the exfoliating bark plays a crucial role in the disease pressure of powdery mildew. Large numbers of chasmothecia and aerially‐dispersed ascospores in spring^17^ are correlated with high disease severity in grapes.^14^ Post‐harvest treatments of the foliage in autumn can effectively reduce the number of chasmothecia or the viability of ascospores.^18^, ^19^, ^20^, ^21^ The treatment of the bark of dormant vines with lime sulphur at the end of winter can also reduce the viability of ascospores.^22^ However, fungicides against powdery mildew are not intended for this kind of application in late autumn or winter in most winegrowing regions in Europe.^23^, ^24^, ^25^ The focus of powdery mildew control is therefore on fungicide treatments during the grape‐growing season, mostly starting before the ascospores can cause the primary infections.^26^ Disease forecast models for fungicide treatments can contribute to a better scheduling of applications and reduction of fungicide use.^11^, ^14^, ^27^, ^28^, ^29^, ^30^, ^31^, ^32^, ^33^, ^34^ Nevertheless, not every model can be used in all wine‐growing regions^11^ because they differ in the underlying biological relationships (ascosporic^32^ or flag shoot‐derived infections^31^) or in the sample methods used to generate these models.^11^ Moreover, the models are not designed for the timing of applications against chasmothecia formation. Fungicide treatments at the end of the season are important to control chasmothecia development,^35^, ^36^, ^37^ especially after the phenological stage, BBCH 79 (‘majority of berries touching’^38^).^14^ Even on vines treated with fungicides after this critical period, up to 350 viable ascospores per vine head were observed on the bark at the time of bud break in Austria,^4^ indicating that chasmothecia control can still be improved.

Several studies have examined the potential of fungicide treatments to reduce chasmothecia formation by repeated applications of active ingredients with a multi‐ or single‐site mode of action during the grape‐growing season.^35^, ^36^, ^37^ However, these studies often do not reflect the fungicide use of wine growers, who use active ingredients with a single‐site mode of action in an alternating application sequence to reduce the risk of resistance of *E. necator*.^39^ Inorganic fungicides, such as sulphur, potassium bicarbonate, and copper compounds, with their multisite mode of action, have a low resistance risk^39^ and may also be used in organic viticulture.^40^

To the best of our knowledge, nothing is known about chasmothecia reduction using synthetic and inorganic fungicides in common spraying sequences of commercial vineyards. In addition, there is a lack of knowledge regarding the effect of netting vines on chasmothecia formation. Nets are increasingly being used in many viticultural areas to protect grapes from birds. It is unclear whether nets favour the formation of chasmothecia due to a possible reduction in ultraviolet B (UVB) radiation because high UVB radiation lowers the germination rate and colony development of grape powdery mildew.^41^

To improve practical recommendations for commercial vineyards, we combined data from spraying sequences in commercial vineyards with an exact experiment that investigated the effects of nets and different fungicide applications at ‘veraison’ (BBCH 81^38^) and immediately before harvest, on chasmothecia formation. The objectives of this study were to (i) evaluate the chasmothecia reduction potential of synthetic and inorganic fungicides, (ii) analyse the effects of the timing of inorganic fungicide applications on chasmothecia formation, and (iii) assess the role of environmental factors (e.g., sun exposure and nets) in chasmothecia formation. By evaluating the effects of inorganic fungicides on chasmothecia, we aimed to identify effective ways to control powdery mildew with minimal fungicide use.

## 2 MATERIAL AND METHODS

### 2.1 Spraying sequences in commercial vineyards

This part of our study was conducted in Burgenland, Austria, in the wine‐growing region of Leithaberg (47°54′ N, 16°41′ E). We selected 32 commercial vineyards, consisting of 16 pairs with organic and integrated management (maximum distance of 200 m between paired vineyards).^42^ The vineyards were planted with various *Vitis vinifera* L. cultivars (Table S1). The spacing of the vines was 2–2.8 m between the rows and 0.9–1.2 m within the rows. The heads of the vines were at a height of 0.9–1.1 m and the vines were Cordon‐ or Guyot‐trained in trellis systems. The age of the vines was 3–70 years. A weather station (OTT Hydromet, Kempten, Germany) was positioned approximately 500 m away from vineyards 3 and 4 (Table S2), and the temperature and precipitation were recorded. The use of plant protection products was left to the personal decision of the respective wine growers, who relied on the farming method (integrated or organic), local practices, recommendations from experts, and their personal experience. In the autumn of 2019 and 2020, wine growers were interviewed using a structured questionnaire to collect information about their fungicide use and other management practices. Out of this information, the number of applied fungicides against *E. necator* (sulphur, copper, potassium bicarbonate, and synthetic fungicides) was calculated for each vineyard from 1 week before BBCH 79,^38^ onwards.

At BBCH 79^38^ (22 July 2019 and 29 July 2020), at least 100 grape clusters per vineyard were inspected visually for the appearance of powdery mildew. The disease severity was determined as the percentage of the total cluster area with symptoms.^43^ The phenological stages were recorded.^38^ After harvest (8 October 2019 and 7 October 2020), 12 leaves (in the cluster zone) were collected from the sunny side (oriented to the south or west) and the shadow side (oriented to the east or north) in each vineyard canopy. Thereafter, the leaves were stored at +4 °C and examined within 7 days. At this temperature, no further chasmothecia development took place.^12^ The number of yellow, brown, and black (mature) chasmothecia was counted in three circles (1.8‐cm diameter) per leaf on the upper side and in three circles on the lower side at 40‐fold magnification and expressed as the number per 100 cm^2^.^44^ The circles were applied at three points along the main veins of the leaves.

### 2.2 Application trial

The trial was conducted in a vineyard in Krems, Austria (48°25′ N, 15°36′ E) in 2018 and 2019, planted with *V. vinifera* ‘Müller Thurgau’ and ‘Grüner Veltliner’. Both cultivars are highly susceptible to *E. necator*.^45^ The inter‐row space was 1.8 and 2.2 m, the in‐row space was 1.1 and 1.2 m, and the heads of the vines were at a height of 1.0 and 1.1 m for ‘Müller Thurgau’ and ‘Grüner Veltliner’, respectively. The vines were Guyot‐pruned with two canes per vine, in a trellis system. A weather station (OTT Hydromet, Kempten, Germany) was positioned at a distance of 100 m from the vineyard and recorded temperature and precipitation at 15‐min intervals.

The vines were treated regularly with fungicides against powdery mildew (Table S3) by the winegrower until BBCH 79^38^ (4 July 2018 and 9 July 2019). The experimental treatments started at ‘veraison’ (BBCH 81,^38^ 20 July 2018 and 5 August 2019) and included 10 vines with three replicates of ‘Müller Thurgau’ and four replicates of ‘Grüner Veltliner’. A backpack, air‐blast sprayer (Stihl SR400; Waiblingen, Germany) was used for all fungicide applications (spray volume, 600 L/ha). Seven different treatments were investigated, with two treatments not using fungicides after BBCH 79^38^: (i) an untreated control (CON) and (ii) a net (mesh 10 × 1.5 mm, green colour, WitaNet; Witasek, Feldkirchen, Austria) fixed on both sides of the canopy (NET). The other treatments received one fungicide application of (iii) copper oxychloride (COP) (0.6 L/ha, Cuprofor® flow; Kwizda Agro, Vienna, Austria), (iv) pyriofenone (PYR) (0.3 L/ha, Kusabi®; Kwizda Agro, Vienna, Austria), (v) quinoxyfen + myclobutanil (QUIN+MYC) (1.1 L/ha, Legend® power; Kwizda Agro, Vienna, Austria), (vi) potassium bicarbonate (POT) (5 kg/ha, Kumar®; Certis, Utrecht, Netherlands) at BBCH 81^38^ or two applications of (vii) potassium bicarbonate (2 × POT) (5 kg/ha, Kumar®; Certis, Utrecht, Netherlands) at BBCH 81^38^ and 1 day before harvest (19 August 2018 and 31 August 2019). These fungicides were chosen because they were widely used in Austrian conventional and organic viticulture in 2018/2019. In all untreated CON plots, chasmothecia development was monitored periodically by microscopic examination from August until leaf‐fall, and in all other treatments, the number of chasmothecia was determined in autumn (20 October 2018 and 14 October 2019). The leaves were therefore stored at +4 °C and examined within 7 days. The number of chasmothecia per leaf (yellow, brown, and black) was counted in three circles (1.8‐cm diameter) on the upper side and in three circles on the lower side at 40‐fold magnification. Circles were applied along the main veins of the leaves. The number of chasmothecia was expressed as the number per 100 cm^2^.^44^

### 2.3 Statistical analyses

R version 3.6.3^46^ and R Studio^47^ were used to perform statistical data analysis and visualisation including the R packages ‘ggplot2’,^48^ ‘stats’,^46^ ‘rstatix’,^49^ ‘car’,^50^ ‘dplyr’,^51^ and ‘exactRankTests’.^52^ Data exploration of the commercial spray sequence datasets and application trial was done by visually identifying outliers, normal distribution, and variance homogeneity.^53^ Furthermore, normal distribution was tested with the Shapiro–Wilk test^54^ and homogeneity was evaluated with Levene's test.^50^, ^55^ The non‐normally distributed data of the commercial spray sequences for 2019 and 2020 were analysed using the two sample Wilcoxon rank‐sum test (equivalent of the Mann–Whitney test),^54^, ^56^ to explore the effects of the management type (organic *vs* integrated) and different frequencies of copper, sulphur, or potassium bicarbonate applications on the number of chasmothecia on vine leaves (*α* = 0.05). More specifically, we analysed the specific frequencies of copper, sulphur, and potassium bicarbonate applications in subsets, but only if the specific frequencies were applied in at least four vineyards (Table 1). Frequencies of applied synthetic fungicides are only shown graphically because the application of synthetic fungicides in one vineyard could consist of a mixture of one or several active ingredients. The non‐normally distributed application trial dataset was also analysed using the two‐sample Wilcoxon rank–sum test (equivalent of the Mann–Whitney test)^54^, ^56^ to test the effect of variety and treatments on chasmothecia development (*α* = 0.05).

**Table 1:** Number of vineyards with respective fungicide application frequencies against *Erysiphe necator* from 1 week before BBCH^38^ 79 in 2019 and 2020 obtained from the commercial spray sequence datasets

Year	Active ingredient	Application frequency (*n*)						*n* (total)
		0	2	3	4	5	6	
2019	Sulphur	6	6	9				21
	Copper	6	7		5			18
	Potassium bicarbonate	22		4				26
	Synthetic	19					6	25
2020	Sulphur	7	5		8			20
	Copper	5	5		9			19
	Potassium bicarbonate	14	4			4		22
	Synthetic	17		4			5	26

*Note*: The number of vineyards (*n*) (subsets) with respective frequencies of copper, sulphur, potassium bicarbonate, and synthetic fungicides were only listed if they were applied in the same frequency in at least four different vineyards.

## 3 RESULTS

### 3.1 Commercial spray sequence datasets

**Figure 1:**
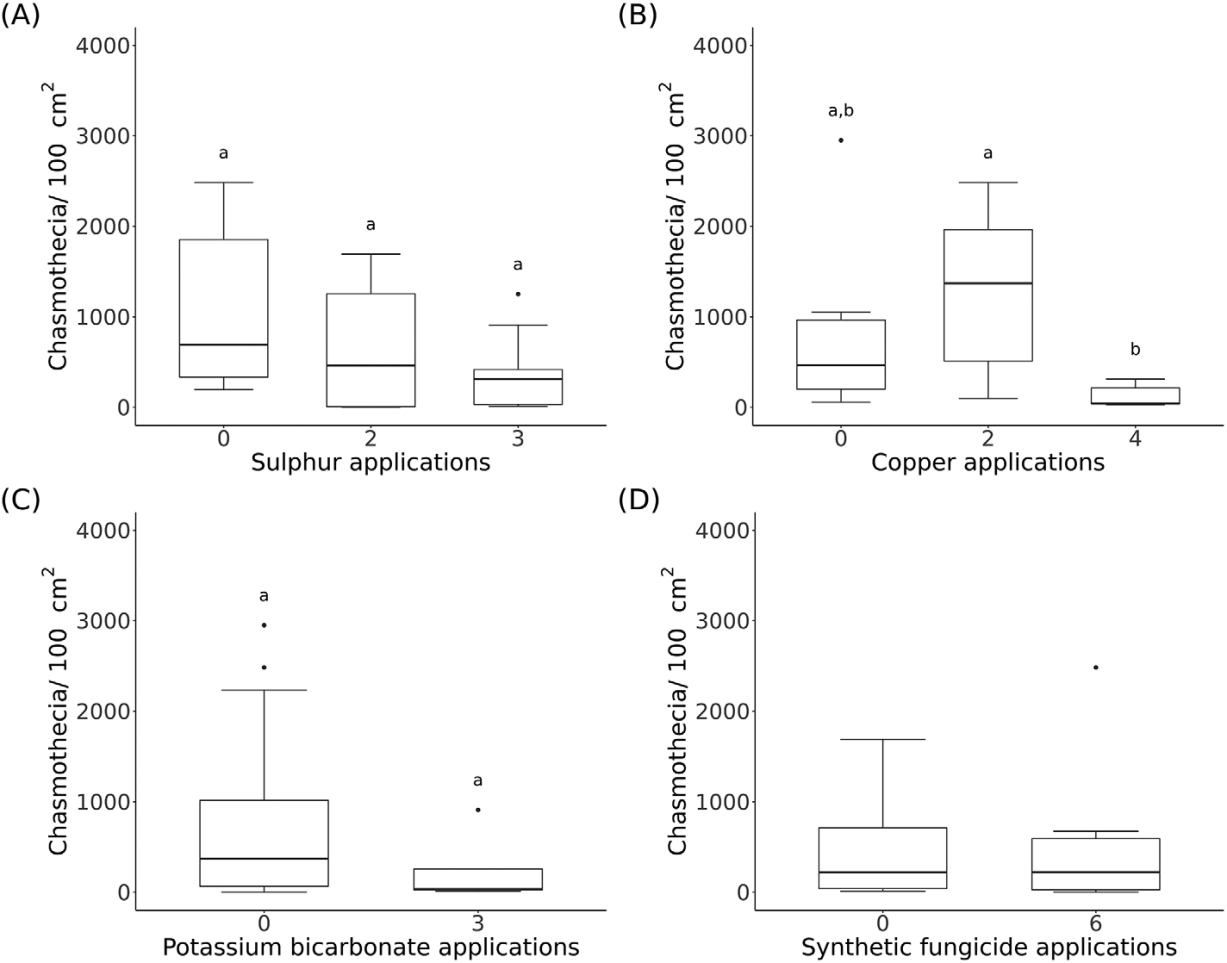
Number of chasmothecia per 100 cm^2^ leaf area from 2019 of the commercial spray sequence datasets in relation to the different application frequencies of the specified active ingredients: (A) sulphur (*n* = 21), (B) copper (*n* = 18), (C) potassium bicarbonate (*n* = 26), and (D) synthetic fungicide (*n* = 25) applications. Significant differences (*P* < 0.05) between different application frequencies of sulphur, copper, and potassium bicarbonate are indicated with different letters above the boxplots.

**Figure 2:**
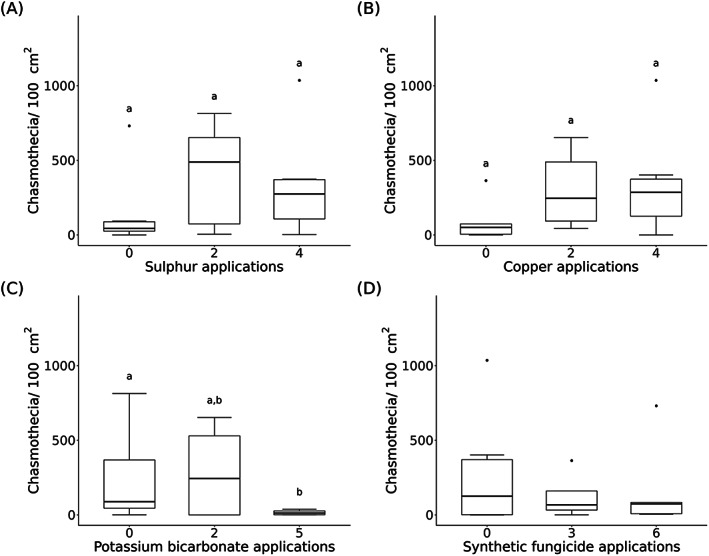
Number of chasmothecia per 100 cm^2^ leaf area from 2020 of the commercial spray sequence datasets in relation to the different application frequencies of the specified active ingredients: (A) sulphur (*n* = 20), (B) copper (*n* = 19), (C) potassium bicarbonate (*n* = 22), and (D) synthetic fungicide (*n* = 26) applications. Significant differences (*P* < 0.05) between different application frequencies of sulphur, copper, and potassium bicarbonate are indicated with different letters above the boxplots.

In 2019, powdery mildew occurred on grapes in 22 of 32 vineyards, whereas in 2020, it only occurred in seven of 31 vineyards. In 2019, chasmothecia were found in 30 of 32 vineyards and in 28 of 31 vineyards in 2020. Therefore, the presence of powdery mildew on grapes during the season does not necessarily correspond to the presence of chasmothecia on the vine leaves at the end of the season in the same vineyard. The number of chasmothecia varied between vineyards in both years (Table 2). Overall, the mean number of chasmothecia on vine leaves was 610 ± 786 standard deviation (SD) chasmothecia per 100 cm^2^ vine leaf area in 2019 and 219 ± 276 chasmothecia in 2020 (Fig. S1). This difference is in accordance with the disease severity of *E. necator* on grape clusters in these vineyards. The mean severity of the disease in all vineyards was 13.4% in 2019 and 11.6% in 2020. The maximum disease severity values were 83.1% in 2019 and 49.8% in 2020 (Table 2). The management type (organic *vs* integrated) of the vineyards (2019: *W* = 134, *P* = 0.831; 2020: *W* = 135.5, *P* = 0.787) (Fig. S2) and the different frequencies of sulphur application did not have any influence on the number of chasmothecia in any of the years (2019: 0 *vs* 2 applications: *W* = 24, *P* = 0.393; 0 *vs* 3: *W* = 40, *P* = 0.144; 2 *vs* 3: *W* = 26.5, *P* = 0.979; 2020: 0 *vs* 2: *W* = 11, *P* = 0.343; 0 *vs* 4: *W* = 14, *P* = 0.12; 2 *vs* 4: *W* = 24, *P* = 0.621) (Figs 1(A) and 2(A)).

**Table 2:** Disease severity of *Erysiphe necator* on grape vine clusters (in %) and total number of chasmothecia per 100 cm^*2*^ vine leaf area in vineyards from the commercial spray sequence datasets in 2019 and 2020

Vineyard number	2019		2020	
	Severity on clusters (%)	Chasmothecia/100 cm^2^ vine leaf area	Severity on clusters (%)	Chasmothecia/100 cm^2^ vine leaf area
1	5.5	349	0	43
2	5	96	0	93
3	0	674	0	489
4	5	2483	0	653
5	5	17	0	1
6	5	709	0	8
7	5	195	0	84
8	83.1	2950	49.8	74
9	5	221	0	731
10	0	1051	0	364
11	34.6	2233	5.5	44
12	0	0	NA	NA
13	0	0	0	23
14	0	4	0	51
15	28.4	56	5	5
16	8.6	911	0	814
17	5.4	41	0	38
18	0	12	0	402
19	50.8	1252	6	246
20	7.9	1371	5	384
21	0	714	0	374
22	5	418	0	286
23	0	32	0	264
24	5	393	5	1036
25	5	214	5	1
26	5	1691	0	370
27	5	40	0	0
28	5	30	0	0
29	0	12	0	0
30	5	910	0	5
31	0	132	0	126
32	5	312	0	2

Abbreviation: NA, not applicable.

Copper and potassium bicarbonate application frequencies had a substantial effect only in one of the 2 years. Four copper applications in 2019 resulted in significantly lower chasmothecia numbers on vine leaves than two copper applications (*W* = 33, *P* = 0.01). A difference between zero and two copper applications (*W* = 15, *P* = 0.445) as well as zero and four copper applications (*W* = 25, *P* = 0.0822) was not observed in 2019. Nevertheless, there was a trend toward lower chasmothecia numbers in vineyards with four copper applications compared to zero (Fig. 1(B)). Different copper applications did not reduce chasmothecia numbers in 2020 (0 *vs* 2: *W* = 5, *P* = 0.15; 0 *vs* 4: *W* = 12, *P* = 0.187; 2 *vs* 4: *W* = 23, *P* = 0.969) (Fig. 2(B)).

**Figure 3:**
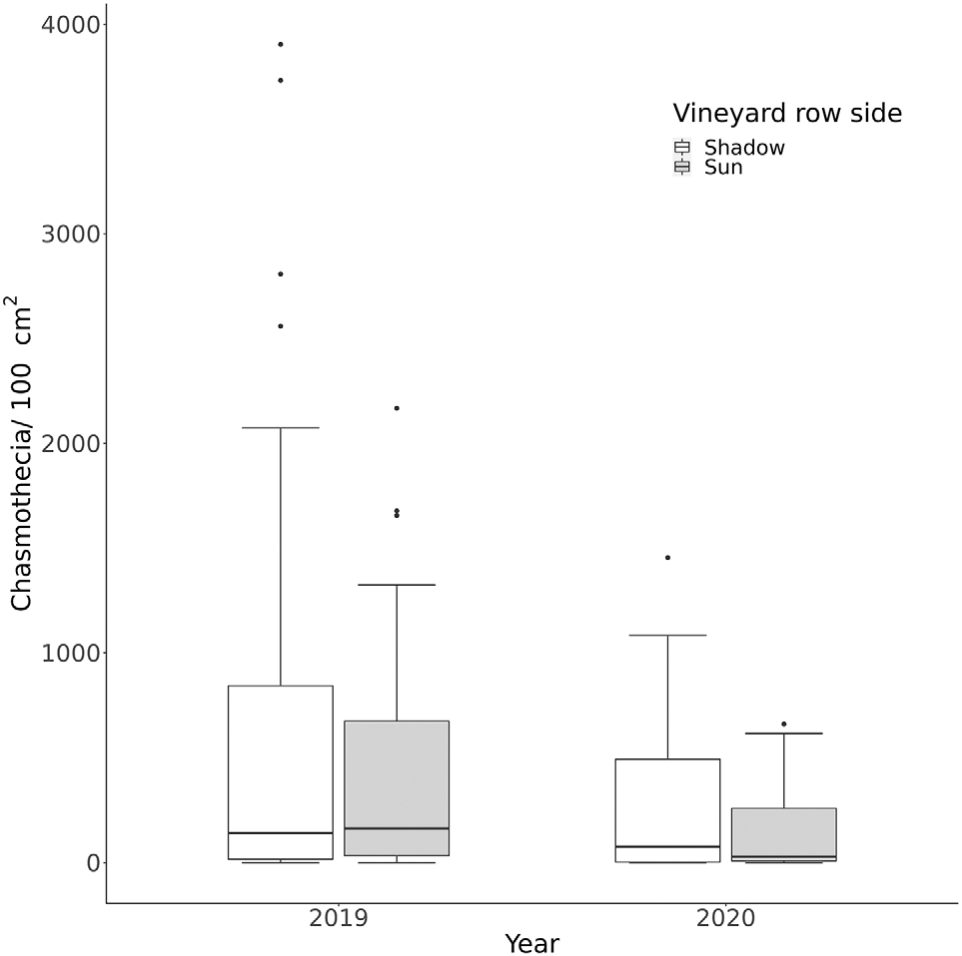
Number of chasmothecia per 100 cm^2^ leaf area in relation to the exposition of the vineyard row (shady or sunny side) of the commercial spray sequence datasets in 2019 and 2020.

In 2020, five potassium bicarbonate applications showed significantly lower chasmothecia numbers than with zero potassium bicarbonate application (*W* = 48.5, *P* = 0.026). Chasmothecia numbers for the other potassium bicarbonate frequencies were similar (0 *vs* 2: *W* = 32, *P* = 0.706; 2 *vs* 5: *W* = 8, *P* = 1) (Fig. 2(C)). In 2019 (which was characterised by high infections of *E. necator)*, there was a general but nonsignificant, negative trend; three applications of potassium bicarbonate seemed to reduce chasmothecia development on vine leaves compared to zero applications (*W* = 62, *P* = 0.218) (Fig. 1(C)). A higher application frequency of synthetic fungicides only seemed to reduce chasmothecia numbers in 2020 (Figs 1(D) and 2(D)). In addition, the data from 2019 and 2020 showed that the vine leaves on the sunny side of the vineyard row tended to develop less chasmothecia than those on the shadowed side (Fig. 3), whereas there was no difference between the lower and upper side of the leaves (Fig. S3). The total precipitation from July to September was twice as high in 2020 (257 mm) than in 2019 (121 mm) (Table S2).

### 3.2 Application trial

**Figure 4:**
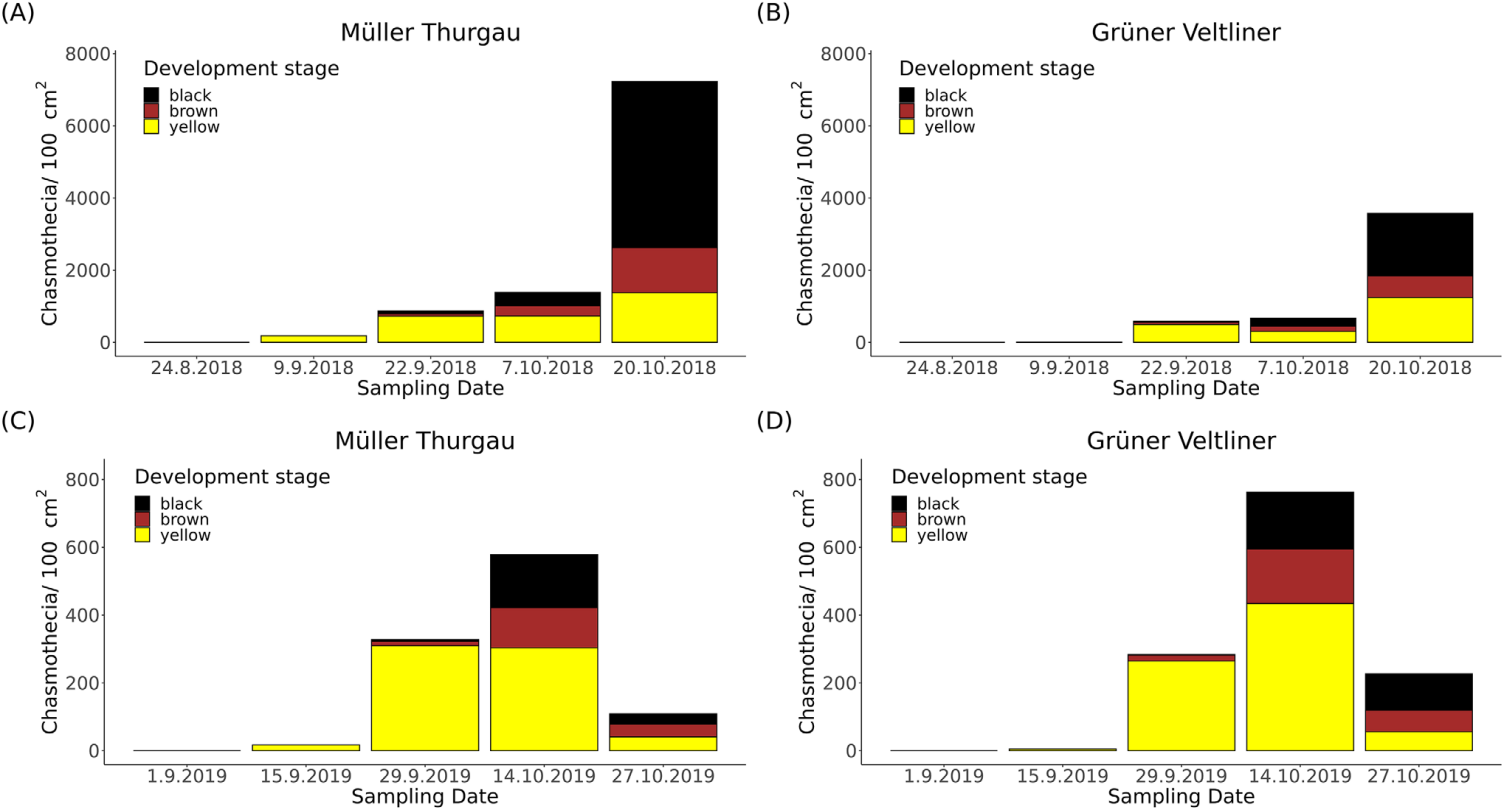
Stacked bar plot from the periodical analysis of the control (CON) from the application trial regarding the development stages (yellow, brown, and black) of the chasmothecia numbers per 100 cm^2^ vine leaf area of the cultivar ‘Müller Thurgau’ in (A) 2018 and (C) 2019, and ‘Grüner Veltliner’ in (B) 2018 and (D) 2019. Differences of the chasmothecia numbers between the 2 years are recognizable on the *y*‐axis scaling.

The development of chasmothecia on vine leaves began in the first half of September in 2018 and 2019. The mean chasmothecia number was 3973 ± 2062 chasmothecia per 100 cm^2^ vine leaf area in 2018, almost eight times higher than that in 2019 (535 ± 332 chasmothecia). The number of yellow chasmothecia usually prevailed until the beginning of (in 2019) or mid‐October (in 2018). After the last sampling date, most chasmothecia were black and were morphologically mature. In 2018, high numbers developed from early to mid‐October, whereas in 2019, chasmothecia numbers declined after mid‐October (Fig. 4). Rainfall events were recorded before the last sampling date in 2019 (Figs S4 and S5).

**Figure 5:**
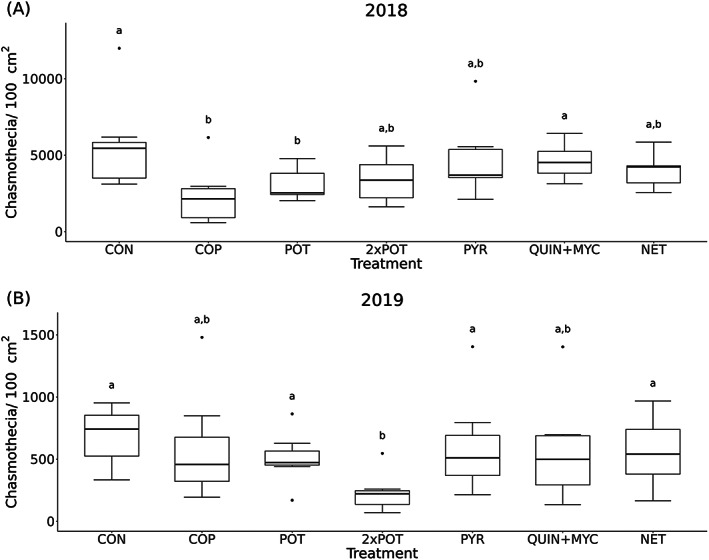
Number of chasmothecia per 100 cm^*2*^ leaf area of the different treatments in (a) 2018 and (b) 2019. Without fungicide application: CON = control, NET = net. With fungicide application: COP = copper oxychloride, PYR = pyriofenone, QUIN+MYC = quinoxyfen + myclobutanil, POT = potassium bicarbonate, 2 × POT = two times potassium bicarbonate. Significant differences (*P* < 0.05) between the treatments of each year are indicated with different letters above the boxplot.

The cultivar ‘Müller Thurgau’ displayed higher chasmothecia numbers on vine leaves than ‘Grüner Veltliner’ in 2018 (*W* = 192, *P* = 0.039). However, in 2019, chasmothecia numbers were higher in ‘Grüner Veltliner’ vines than ‘Müller Thurgau’ (*W* = 407, *P* = 0.021) (Fig. S6). The chasmothecia numbers were only reduced by COP treatment compared with CON (*W* = 44, *P* = 0.011) and QUIN+MYC (*W* = 6, *P* = 0.017). This was also the case for POT compared with CON (*W* = 41, *P* = 0.037) and QUIN+MYC (*W* = 8, *P* = 0.037) in 2018 (Fig. 5(A)). In 2019, only 2 × POT reduced chasmothecia compared to CON (*W* = 47, *P* = 0.002), POT (*W* = 41, *P* = 0.037), PYR (*W* = 7, *P* = 0.026), and NET (*W* = 7, *P* = 0.026) (Fig. 5(B)).

## 4 DISCUSSION

In this study, we examined the potential of several fungicides in different commercial vineyards and in an application trial to reduce chasmothecia development. Chasmothecia numbers in the organic and integrated commercial vineyards were similar. Furthermore, inorganic fungicides controlled chasmothecia formation from the ‘majority of berries touching’ (BBCH 79^38^) onwards and were as effective as equal or higher applications of synthetic fungicides in the commercial spray sequence datasets. Potassium bicarbonate was especially effective in reducing the number of chasmothecia.

In 2019, chasmothecia numbers in vineyards with spray sequences containing four copper or three potassium bicarbonate applications were similar to spray sequences with synthetic fungicides. In 2020, five potassium bicarbonate applications resulted in chasmothecia numbers comparable to those of synthetic fungicide spray sequences. This is in accordance with earlier findings where potassium bicarbonate and copper oxychloride, as well as synthetic fungicides, demonstrated effective reduction of the viability of ascospores in chasmothecia.^21^ However, copper compounds are not registered as pesticides against powdery mildew in many countries^23^, ^24^, ^25^, ^57^ because their efficiency is considered to be too low to provide adequate control of powdery mildew disease.^1^ Several studies have demonstrated that the reduction in chasmothecia numbers using different synthetic fungicides,^35^, ^36^, ^37^ sulphur,^35^, ^36^ and the biocontrol agent *Ampelomyces quisqualis* Ces.^19^ as in‐season treatments (applied between BBCH 07–89^38^) is rather low. Unfortunately, in some of these studies, the alternation of active ingredients was not considered; the same fungicides were sprayed repeatedly during the season.^35^, ^36^, ^37^ This does not reflect the common application practice in viticulture, which is characterised by frequent changes in active ingredients for resistance management. Based on our study, it must also be considered that fungicides with the same and/or different modes of action can be applied at various rates, times, and in variable intervals, resulting in different disease control levels.^39^ This was especially the case in the commercial spray sequence datasets. Weather conditions were relatively similar for all sampled vineyards in the same wine‐growing region, but different timing of applications and the associated weather conditions might also have influenced chasmothecia formation. Nevertheless, the results that incorporate commercial spray sequences reflect *in situ* conditions in commercial vineyards more appropriately.

The incidence of powdery mildew on grapes at the ‘majority of berries touching’ stage did not correspond with the amount of chasmothecia after harvest in the same vineyards. The number of chasmothecia on leaves depends on the severity of powdery mildew on vine leaves in the previous autumn.^14^, ^37^, ^58^ Reducing the number of chasmothecia in autumn can lower the disease severity in the subsequent season.^14^, ^19^, ^22^ This fact indicates the importance of controlling powdery mildew from the ‘majority of berries touching’ stage onwards for an effective reduction in chasmothecia formation the following year. The current study demonstrated a notable reduction in chasmothecia numbers on vine leaves in 2019 using several applications of sulphur, copper, or potassium bicarbonate in commercial spay sequences after the ‘majority of berries touching’ stage. This is in accordance with another study which demonstrated that the earlier cessation of sulphur and kresoxim‐methyl applications in the growing season resulted in an increase in chasmothecia formation on the vine leaves.^14^ This emphasises the comparable efficiency of inorganic and synthetic fungicides. One reason could be related to the systemic properties of synthetic fungicides within the plant tissue.^39^ Most of the synthetic fungicides have systemic properties, resulting in movement within the plant tissues.^39^ In contrast, inorganic fungicides, such as sulphur and copper, have a low mobility within plant tissues or remain for a specific time period on the leaf surface.^39^ The latter disadvantage is ameliorated at the end of the main growth period because the leaf area increase is low.^59^

That copper was effective in the first season and potassium bicarbonate in the second, can be explained by the different modes of action of both fungicides. Copper is mainly prophylactic, whereas potassium bicarbonate is eradicative with no residual prophylaxis.^39^ Possibly, powdery mildew colonies had not yet been formed when potassium bicarbonate was applied in most vineyards in 2019. In 2020, the lower reduction in chasmothecia by copper could have been affected by twice the total precipitation from July to September 2020 compared to 2019; copper ions can easily be washed off by rain.^60^

The considerable differences in chasmothecia numbers between the years investigated in our study has also been observed in commercial vineyards in Trentino,^58^ where environmental conditions were a major factor influencing chasmothecia numbers on leaves. A low UVB radiation in this context is known to favour disease development,^41^ which explains the trend from the commercial spray sequence datasets in which chasmothecia numbers were slightly higher on the shady side of the canopy. A study also showed that a canopy of netted vines resulted in a reduced light level in the canopy.^61^ Contrary to our expectations, the NET treatment did not substantially increase chasmothecia numbers compared to the control in the application trial.

The results from the application trial showed that the COP (copper treatment) and POT (potassium bicarbonate treatment) treatments were able to substantially reduce the number of chasmothecia in 2018, when grapes ripened earlier than usual. Furthermore, the 2 × POT treatment led to a substantial reduction in chasmothecia numbers in 2019. Potassium bicarbonate showed a consistent reduction in both years, whereas copper was effective only in 2018. Consistent with the previously mentioned commercial spray sequence datasets, environmental conditions may be responsible for the inconsistent effect of copper.^60^ Another reason could be the later onset of chasmothecia formation in 2019 than in 2018. Post‐harvest treatments can be an effective alternative for reducing chasmothecia numbers in years with an early harvest.^21^ However, post‐harvest applications (with the exception of a few copper fungicides) are not intended in viticulture in many Central European countries.^23^, ^24^, ^25^

Replacing synthetic fungicides with copper and potassium bicarbonate starting at ‘majority of berries touching’ stage until the end of the season minimises the risk of relevant residues of active ingredients on grapes and in wines.^62^ As previously mentioned, potassium bicarbonate and copper compounds have some advantages as multisite inhibitors against powdery mildew, which is in contrast to many synthetic fungicides.^39^ Therefore, the substitution of single‐site inhibitors using a few applications of copper compounds and potassium bicarbonate, without any loss of efficacy against chasmothecia formation, can contribute to lowering the probability of the emergence of resistant strains. Furthermore, two diseases (downy and powdery mildew) were controlled using one active ingredient. This contributes to the EU ‘Farm to Fork’ goal of halving chemical pesticide use by 2030.^63^ A disadvantage of the intensive use of the inorganic fungicides is that they can have negative impacts on natural enemies against pests on vines^42^, ^64^ and soil biota.^39^, ^65^ Furthermore, wine aroma components, such as volatile thiols, can be reduced by the intensive application of copper in a few cultivars.^66^ However, these negative effects of copper can be reduced when it is applied in fewer applications only at the end of the season. This strategy complies with the current requirements for copper reduction and the maximum limits permissible in plant protection.^60^ It is necessary to mention here that a reduction in copper applications can increase the potential disease pressure of powdery mildew in organic vineyards.

## 5 CONCLUSION

The prevention of *E. necator* infestation is crucial for reducing quality losses in grapes caused by this pathogen. Warm weather conditions are especially conducive to a high powdery mildew infection rate and chasmothecia formation. It is also very important to reduce the amount of chasmothecia as the primary inoculum at the end of the grape‐growing season, which could possible induce a lower infection in the subsequent seasons. This goal can be achieved using synthetic or inorganic fungicides; the latter can also be applied by organic wine growers. Two applications with potassium bicarbonate or one with copper can effectively lower the number of chasmothecia. Both inorganic fungicides should be sprayed as late as possible during the season, before harvest. This is critical because postharvest fungicide applications to reduce chasmothecia numbers in vineyards are not included in the registration of plant protection products in Central Europe, therefore wine growers should focus on late, in‐season applications as a means of reducing chasmothecia numbers.

## AUTHOR CONTRIBUTIONS

Conceptualization: MR and SM. Methodology: MR. Sample collection: MR and FH. Data analysis and validation: SM. Investigation: SM and MR. Resources: SW and SS. Data curation: SM. Writing—original draft preparation: MR and SM. Writing—review and editing: SS, SW and FH. Visualization: SM. Supervision: SS and SW. Project administration: SW. Funding acquisition: SW. All authors have read and agreed to the published version of the manuscript.

## CONFLICTS OF INTEREST

The authors declare no conflict of interest.

## Supporting information


**Data S1:** Supporting Information.

## Data Availability

The data that support the findings of this study are openly available in Zenodo at https://zenodo.org/record/6472046#.Y8_SDIQxkuU.
