# Supplementary material for: Efficiency of inorganic fungicides against the formation of Erysiphe necator chasmothecia in vineyards
Source: Pest Manag Sci. 2023 Apr 14;79(9):3080–9. doi: 10.1002/ps.7487 (PMC10952470; doi:10.1002/ps.7487)
Supplement: Supplementary file 1 — Data S1: Supporting Information. [file PS-79-3080-s001.docx]

**Supplementary information for the article:**

**Efficiency of inorganic fungicides against the formation of *Erysiphe necator* chasmothecia in vineyards**

Stefan Möth*‘, Markus Redl*, Silvia Winter, Florian Hüttner, Siegrid Steinkellner

University of Natural Resources and Life Sciences, Vienna, Department of Crop Sciences, Institute of Plant Protection, Gregor-Mendel-Strasse 33, 1180 Vienna, Austria

*equally contributing

‘corresponding author: University of Natural Resources and Life Sciences, Vienna, Department of Crop Sciences, Institute of Plant Protection, Gregor-Mendel-Strasse 33, 1180 Vienna, Austria. E-mail: stefan.moeth@boku.ac.at

Table S1: List of planted vine cultivars in the vineyards of the commercial spray sequence datasets.

| Vineyard number | Vine cultivar |
| --- | --- |
| 1 | Cabernet Sauvignon |
| 2 | Blaufränkisch |
| 3 | Blaufränkisch |
| 4 | Blaufränkisch |
| 5 | Cabernet Sauvignon |
| 6 | Zweigelt |
| 7 | Grüner Veltliner |
| 8 | Welschriesling |
| 9 | Grüner Veltliner |
| 10 | Zweigelt |
| 11 | Chardonnay |
| 12 | Sauvignon Blanc |
| 13 | Zweigelt |
| 14 | Blaufränkisch |
| 15 | Furmint |
| 16 | Blauer Burgunder |
| 17 | Blauer Burgunder |
| 18 | Grüner Veltliner |
| 19 | Chardonnay |
| 20 | Blaufränkisch |
| 21 | Weißer Burgunder |
| 22 | Grüner Veltliner |
| 23 | Zweigelt |
| 24 | Blaufränkisch |
| 25 | Weißer Burgunder |
| 26 | Chardonnay |
| 27 | Welschriesling |
| 28 | Welschriesling |
| 29 | Weißer Burgunder |
| 30 | Blaufränkisch |
| 31 | Blaufränkisch |
| 32 | Blauer Burgunder |

Table S2: Average temperature and total precipitation per month in the study area of the commercial spray sequence datasets in 2019 and 2020.

| Month | 2019 | | 2020 | |
| --- | --- | --- | --- | --- |
|  | Temperature (°C) | Precipitation (mm) | Temperature (°C) | Precipitation (mm) |
| January | 0.8 | 47.8 | 0.9 | 23.0 |
| February | 5.1 | 18.8 | 6.9 | 20.0 |
| March | 9.4 | 23.2 | 7.7 | 37.0 |
| April | 12.7 | 13.2 | 13.4 | 4.4 |
| May | 13.3 | 149.0 | 14.9 | 58.0 |
| June | 24.0 | 25.2 | 19.6 | 92.0 |
| July | 23.0 | 19.4 | 21.9 | 35.0 |
| August | 23.4 | 50.6 | 22.8 | 135.8 |
| September | 17.3 | 51.4 | 17.6 | 86.2 |
| October | 12.5 | 20.6 | 11.4 | 118.6 |
| November | 8.1 | 53.6 | 5.9 | 10.8 |
| December | 3.6 | 37.4 | 2.8 | 47.0 |

Table S3: Fungicides use in the application trial against Erysiphe necator in the cultivar ‘Müller Thurgau’ and ‘Grüner Veltliner’ in Krems until BBCH 79 (‘majority of berries touching’^1^) in 2018 and 2019.

| Year | Week | Phenology  (BBCH) | Product | Active ingredient | Application rate  (kg or l/ha) |
| --- | --- | --- | --- | --- | --- |
| 2018 | 16 | 9 | Thiovit Jet | Sulphur | 2.30 |
|  | 19 | 16 | Thiovit Jet | Sulphur | 0.60 |
|  |  |  | Flint | Trifloxystrobin | 0.07 |
|  | 21 | 57 | Thiovit Jet | Sulphur | 0.75 |
|  |  |  | Topas | Penconazole | 0.13 |
|  | 22 | 69 | Thiovit Jet | Sulphur | 1.20 |
|  |  |  | Sercadis | Fluxapyroxad | 0.16 |
|  |  |  | Karathane Gold | Meptyldinocap | 0.10 |
|  | 24 | 75 | Thiovit Jet | Sulphur | 1.20 |
|  |  |  | Legend | Quinoxyfen | 0.20 |
|  |  |  | Karathane Gold | Meptyldinocap | 0.12 |
|  | 27 | 79 | Thiovit Jet | Sulphur | 1.12 |
|  |  |  | Collis | Boscalid | 0.37 |
|  |  |  |  | Kresoxim-methyl |  |
| 2019 | 16 | 9 | Thiovit Jet | Sulphur | 2.30 |
|  | 20 | 15 | Thiovit Jet | Sulphur | 0.50 |
|  |  |  | Flint | Trifloxystrobin | 0.05 |
|  | 22 | 57 | Thiovit Jet | Sulphur | 0.60 |
|  |  |  | Topas | Penconazole | 0.10 |
|  | 24 | 71 | Thiovit Jet | Sulphur | 1.05 |
|  |  |  | Sercadis | Fluxapyroxad | 0.18 |
|  | 26 | 73 | Thiovit Jet | Sulphur | 1.20 |
|  |  |  | Legend power | Quinoxyfen | 1.00 |
|  |  |  |  | Myclobutanil |  |
|  |  |  | Karathane Gold | Meptyldinocap | 0.13 |
|  | 28 | 79 | Thiovit Jet | Sulphur | 0.98 |
|  |  |  | Collis | Boscalid | 0.32 |
|  |  |  |  | Kresoxim-methyl |  |

**Reference**

1 Lorenz DH, Eichhorn KW, Bleiholder H, Klose R, Meier U, and Weber E, Phänologische Entwicklungsstadien der Weinrebe (Vitis vinifera L. ssp. vinifera). Codierung und Beschreibung der erweiterten BBCH‐Skala, Vitic Enol Sci 49:66–70 (1994).


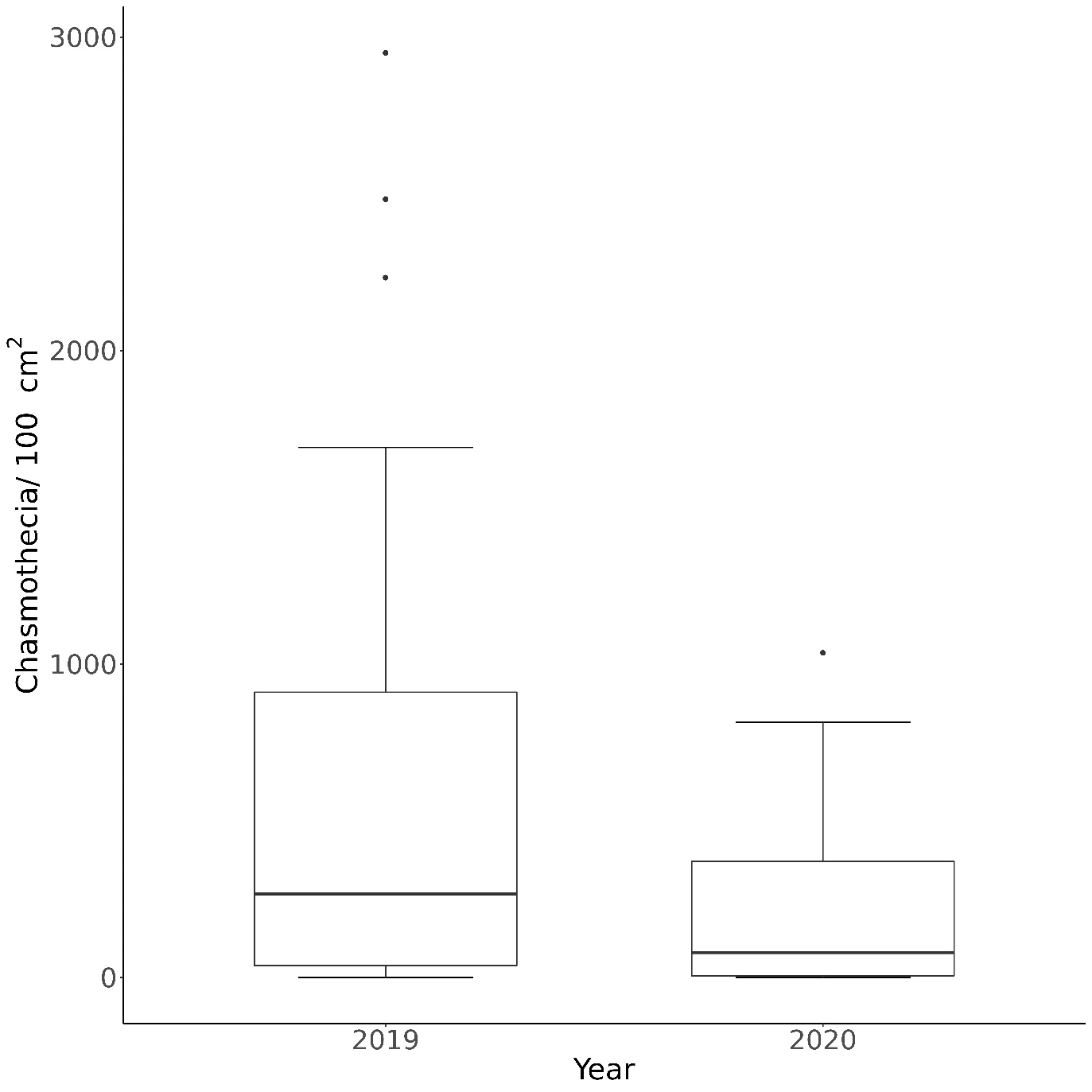


Figure S1: Number of chasmothecia per 100 cm² leaf area in 2019 and 2020 of the commercial spray sequence datasets.


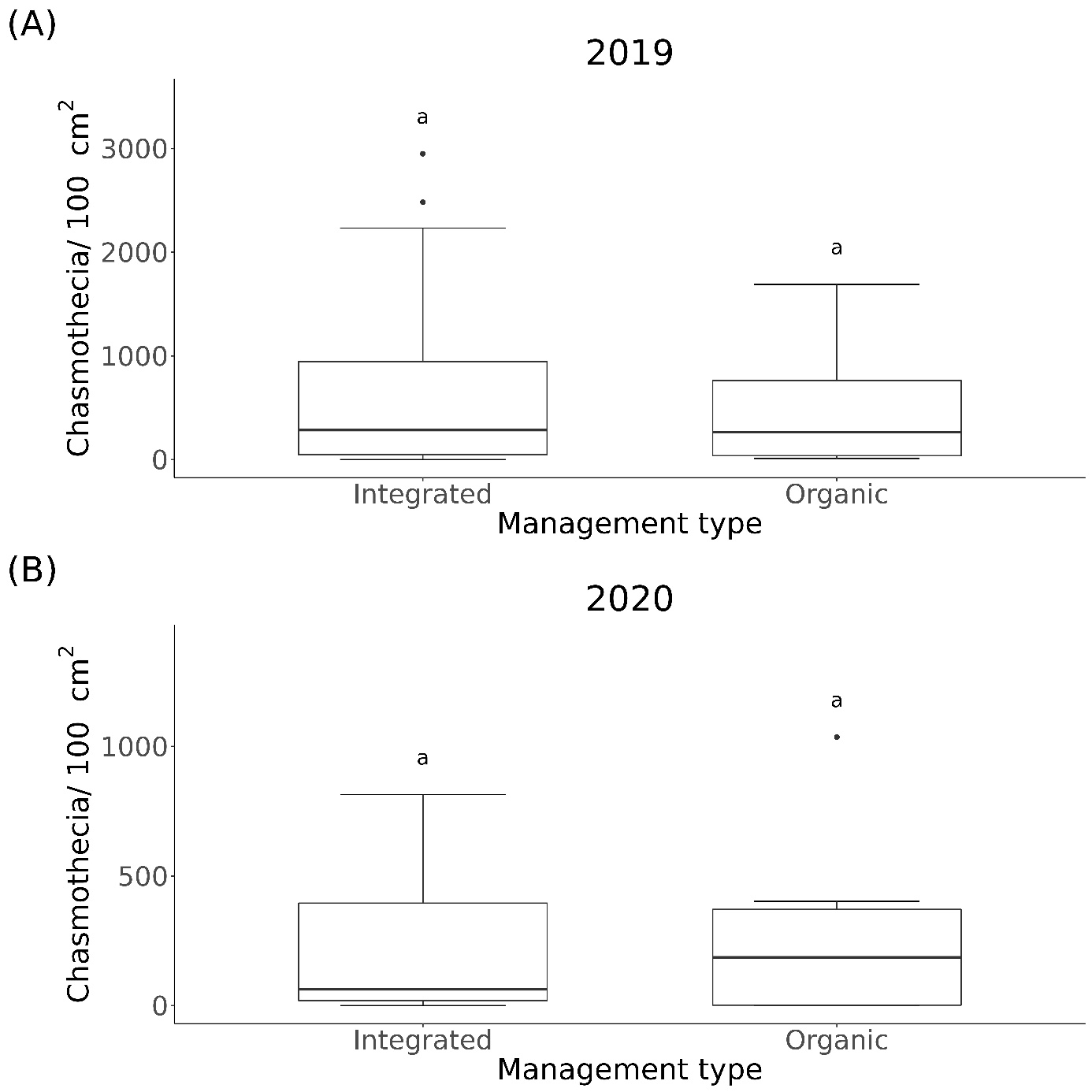


Figure S2: Number of chasmothecia per 100 cm² leaf area in relation to the different management types (organic versus integrated) in (A) 2019 and (B) 2020 of the commercial spray sequence datasets. Y-axis scaling differs between 2019 and 2020. Significant differences (P < 0.05) between the management types in each year are indicated by different letters above boxplots.


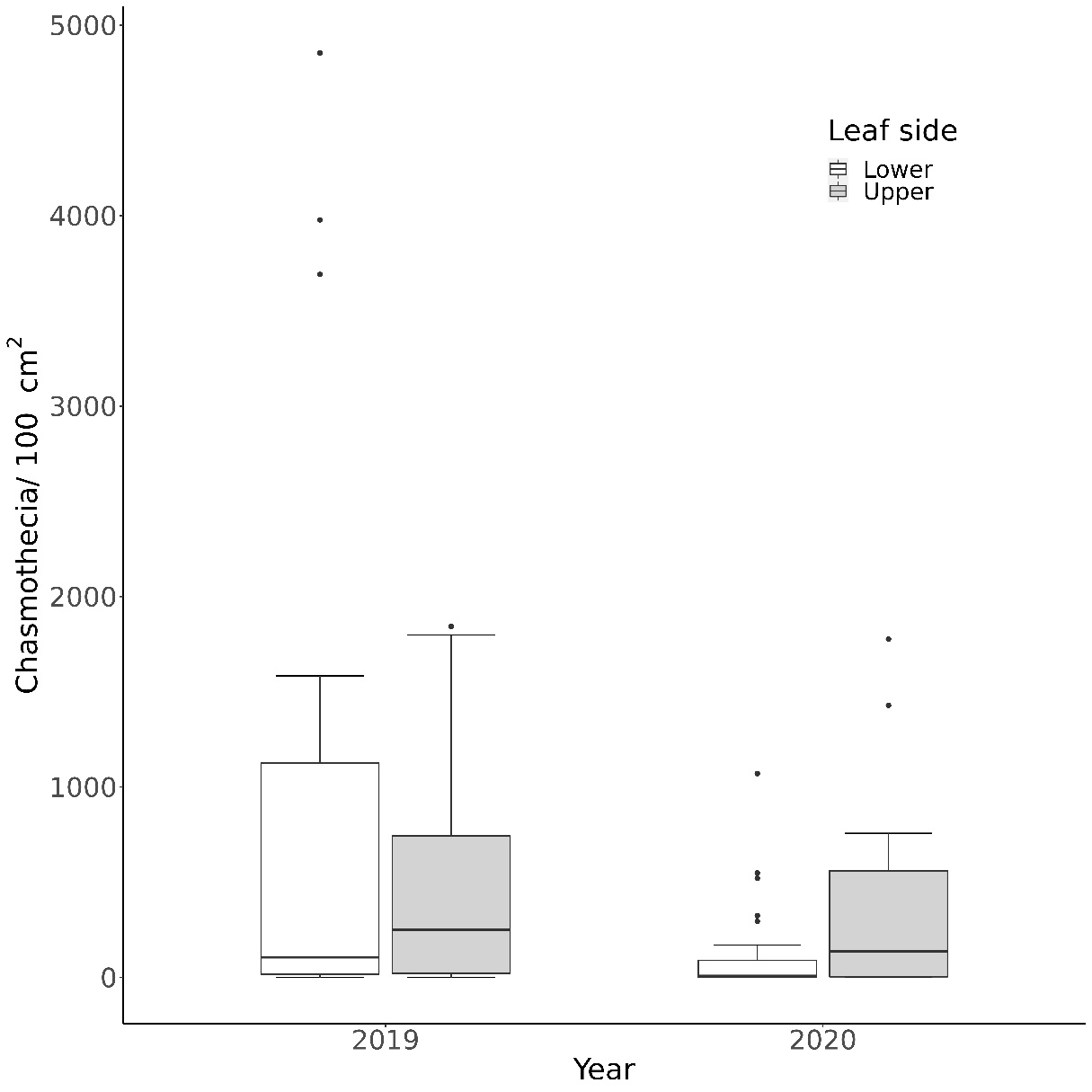


Figure S3: Number of chasmothecia per 100 cm² leaf area of the commercial spray sequence datasets due to different densities on the vine leave lower or upper side in 2019 and 2020.


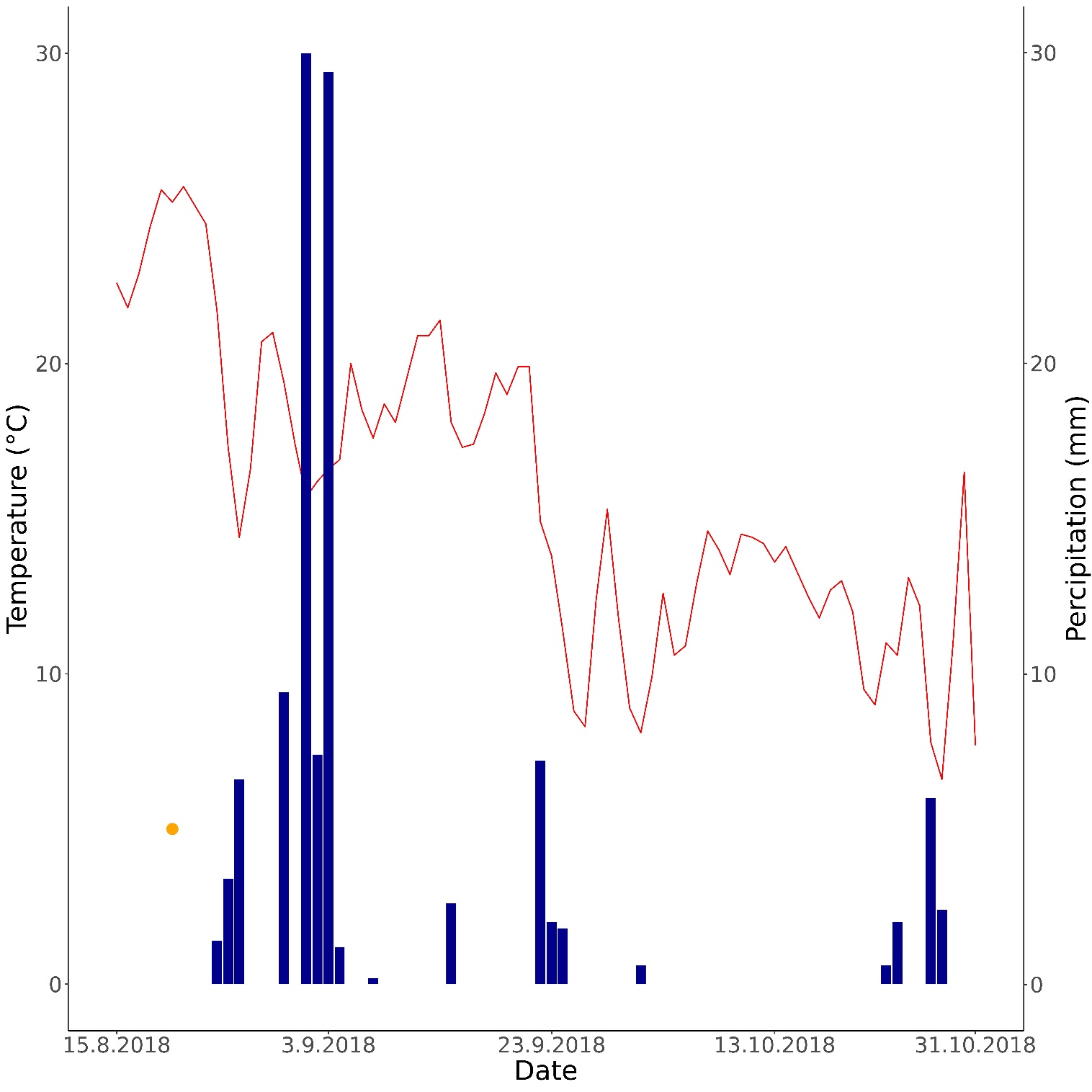


Figure S4: Weather data of the application trial in 2018. The mean temperature in °C is depicted as red line and precipitation in mm as blue bars from 15 August to 31 October. The orange dot indicates the date of harvest on 20 August, 2018.


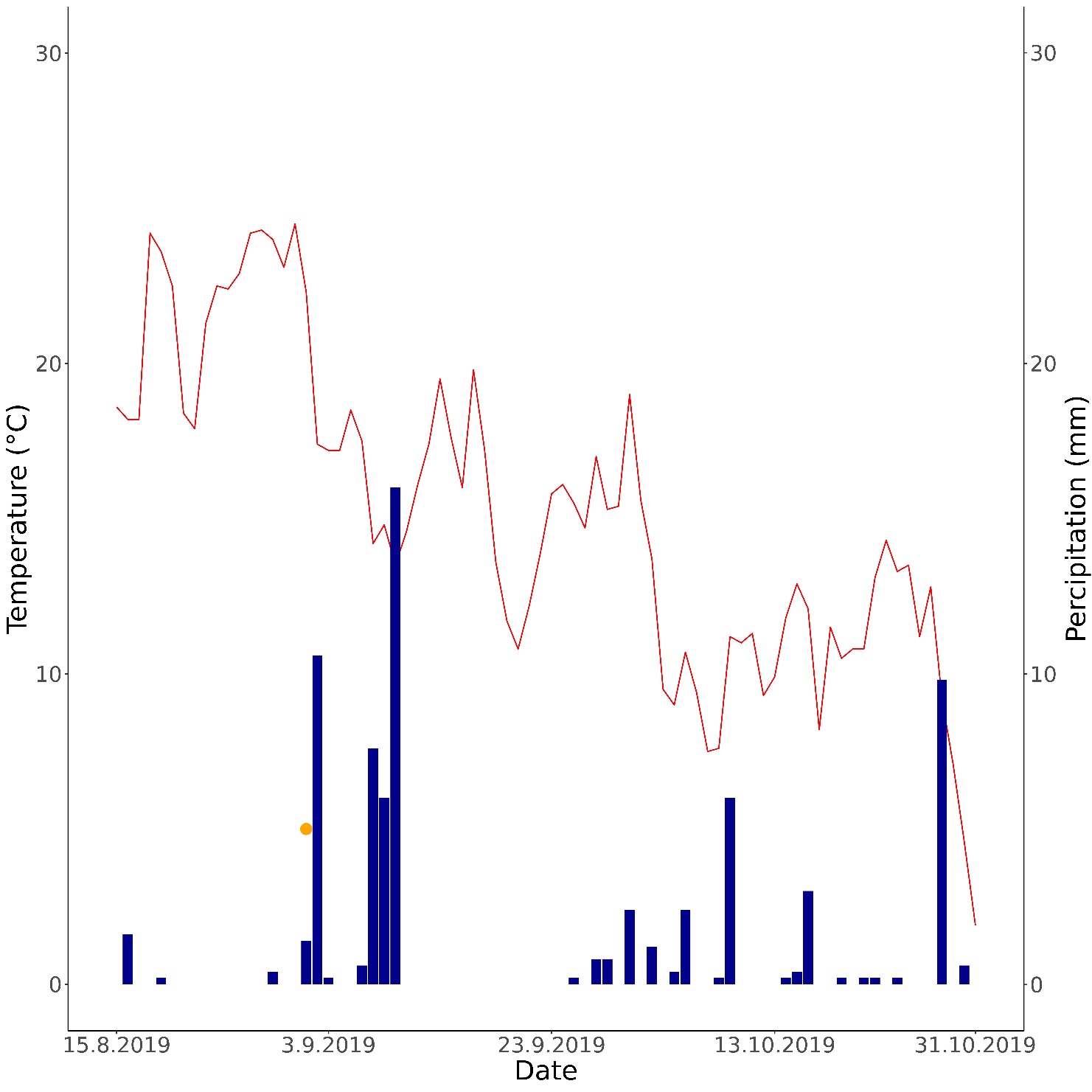


Figure S5: Weather data of the application trial in 2019. The mean temperature in °C is depicted as red line and precipitation in mm as blue bars from 15 August to 31 October. The orange dot indicates the date of harvest on 1 September, 2019.


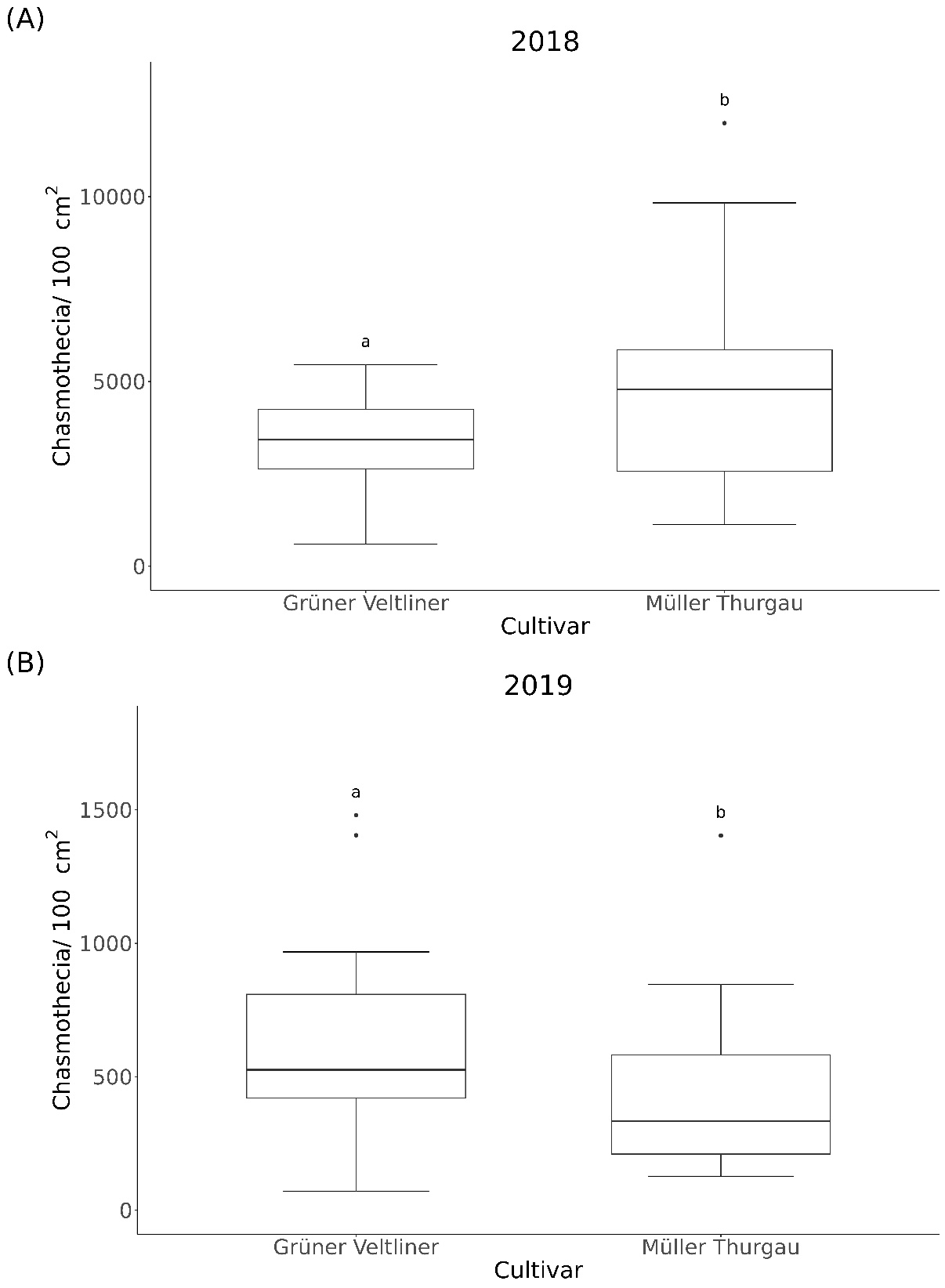


Figure S6: Number of chasmothecia per 100 cm² leaf area of the two different vine cultivars ‘Grüner Veltliner’ and ‘Müller Thurgau’ in (A) 2018 and (B) 2019 of the application trial. Significant differences (P < 0.05) between the cultivars of each year are indicated with different letters above the boxplots.
